# Full-Thickness Macular Hole Formation after Internal Limiting Membrane Peeling: Beware the “*Omega Sign*”

**DOI:** 10.1155/2016/9858291

**Published:** 2016-09-25

**Authors:** Robert A. Sisk, Okan Toygar

**Affiliations:** ^1^Cincinnati Eye Institute, Cincinnati, OH, USA; ^2^Department of Ophthalmology, University of Cincinnati School of Medicine, Cincinnati, OH, USA; ^3^Department of Ophthalmology, Bahcesehir University Faculty of Medicine, Istanbul, Turkey

## Abstract

*Purpose*. To introduce a clinical sign on spectral domain optical coherence tomography (SDOCT), which may indicate high risk for full-thickness macular hole formation after internal limiting membrane (ILM) peeling.* Methods*. The preoperative SDOCT images of two patients—one with multilaminar hemorrhage from ruptured retinal artery macroaneurysm and one with serous retinal detachment and severe macular schisis from optic pit maculopathy—who developed full-thickness macular hole (FTMH) after ILM peeling were evaluated retrospectively.* Results*. On the preoperative SDOCT images of both patients there was a thin bridge of tissue on either side of the foveal center with an outer retinal defect. The photoreceptors were displaced laterally away from the foveal center to create an “*omega-*” shaped configuration of the remaining tissue.* Conclusion*. “*Omega-*” shaped configuration on SDOCT may represent a higher risk of FTMH following ILM peeling. Vitreoretinal surgeons may wish to consider this sign in the process of their surgical decision making.

## 1. Introduction

Peeling of the internal limiting membrane (ILM) has become a preferred technique in the surgical treatment of miscellaneous macular disorders [1]. However, removal of the ILM over the fovea induces permanent anatomical changes, and the consequences are not fully understood [2, 3]. Histologic examinations of excised ILM specimens contained fragments of Müller cells. Gass hypothesized that the pathogenesis of idiopathic full-thickness macular hole (FTMH) formation was disruption of the Müller cell cone (MCC) [4], which may occur iatrogenically during ILM peeling.

In this report we introduce a clinical sign on spectral domain optical coherence tomography (SDOCT) which may indicate high risk for FTMH formation after ILM peeling.

## 2. Case  1

**Figure 1:**
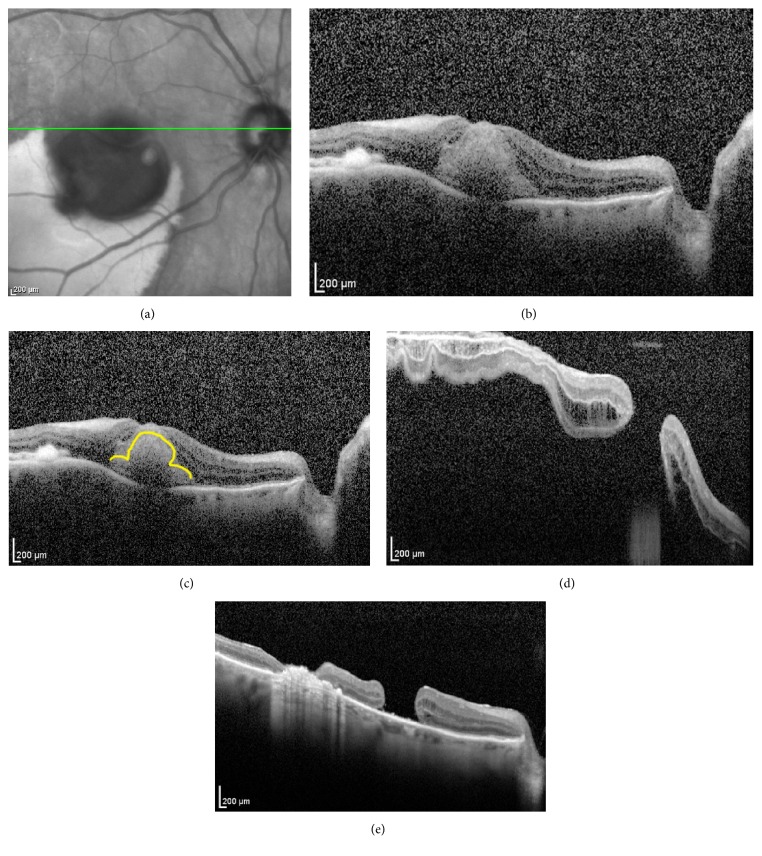
(a) Infrared reference image and (b) foveal spectral domain optical coherence tomography (SDOCT) raster scan of the right macula. (c) Subretinal hemorrhage displaced outer retinal tissue laterally to produce an “*omega-*” shaped contour for the outer retina superimposed as yellow on same SDOCT raster scan. (d) Foveal SDOCT raster scan after PPV with subretinal tissue plasminogen activator and ILM peeling that included the fovea, which had resulted postoperatively in macular hole with serous and rhegmatogenous retinal detachment that failed to clear spontaneously. (e) Foveal SDOCT raster scan five months postoperatively demonstrates persistent macular hole and temporal sealed retinotomy with underlying subretinal hyperreflective material and transmission due to retinal atrophy.

A 91-year-old hypertensive woman presented with sudden onset of central scotoma and 20/200 vision due to multilaminar hemorrhage from ruptured retinal artery macroaneurysm OD. Subretinal blood extended beneath the fovea. Arthritis limited face-down positioning for pneumatic displacement, so pars plana vitrectomy (PPV) and subretinal tissue plasminogen activator injection were performed. The ILM was peeled to liberate trapped sub-ILM hematoma.  Figure 1 demonstrates the preoperative and postoperative SDOCT images. Postoperatively she had localized serous and rhegmatogenous retinal detachment (RD) from FTMH. The patient refused silicone oil and underwent PPV with temporal relaxing retinotomy and 16% C3F8 tamponade. Five months later FTMH was persisting and the visual acuity was 20/200.

## 3. Case  2

**Figure 2:**
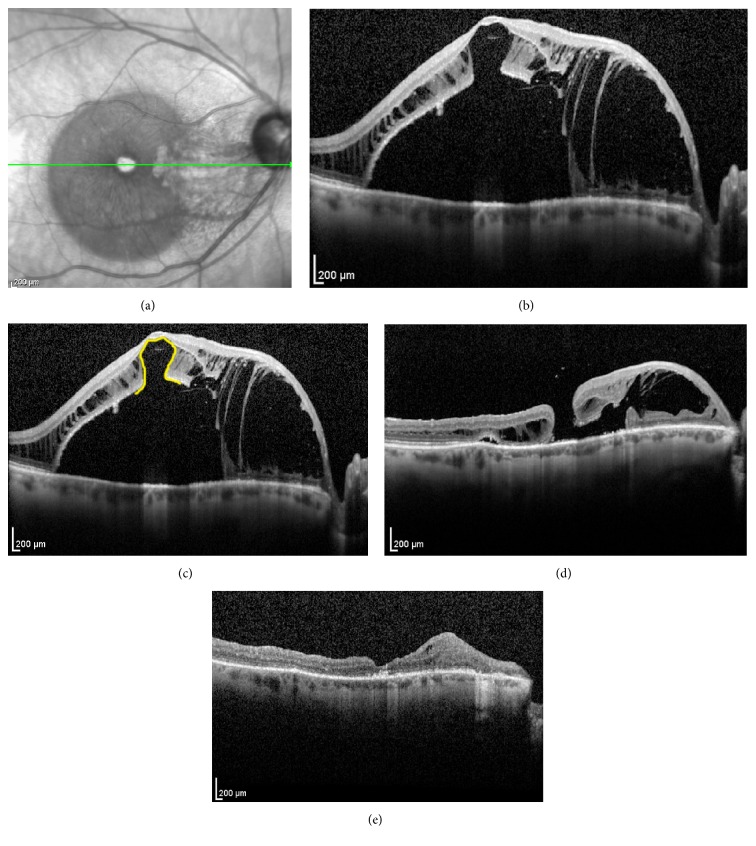
(a) Infrared reference image and (b) foveal SDOCT raster scan of the right macula. Infrared imaging showed hyperintensity within areas of nasal macular schisis within Henle's layer and hypointensity in areas temporally with serous retinal detachment. The foveal center appears hyperintense from transmission defect. Schisis involved the photoreceptor and outer plexiform layers with intact nerve fiber layer. (c) Lateral displacement of the photoreceptors produced the* “omega sign”* on SDOCT, highlighted in yellow, indicating that only the Müller cell cone was bridging the foveola. (d) SDOCT foveal raster scan six weeks after PPV with ILM peeling shows FTMH 5 contiguous with resolving schisis cavity nasally. (e) One year later, after silicone oil was removed, the FTMH remained closed; schisis had resolved. Thinning and disorganization linger among layers previously affected by severe schisis.

A 66-year-old woman presented with serous RD and severe macular schisis from optic pit maculopathy OD. Her visual acuity was finger counting and she complained of a central scotoma for several years. Pars plana vitrectomy, ILM peeling, and long-acting gas tamponade were performed with face-down positioning postoperatively.  Figure 2 demonstrates the preoperative and postoperative SDOCT images. Six weeks after the surgery visual acuity improved to 20/400 but FTMH occurred, which was closed with a following PPV and silicone oil tamponade. One year later, after silicone oil was removed, the FTMH remained closed, schisis had resolved, and visual acuity improved to 20/125.

## 4. Discussion

Both cases demonstrate the necessity of the MCC for structural integrity of the fovea when a prominent outer retinal defect and localized serous or hemorrhagic RD are present. The bridging tissue over the foveola and lateral displacement of the photoreceptor band produced a horseshoe-shaped configuration that we termed the “*omega sign.*” Preoperative recognition of this sign on SDOCT should warn surgeons against ILM peeling over the fovea, which may result in FTMH. Although the report of the bridging tissue is not novel, the risk of creating a macular hole by peeling it has not been well described. The more common situation for the bridging tissue is tractional foveoschisis in myopic eyes. In the study conducted by Kim et al., among the 17 eyes, macular hole was found in two eyes, where one of these eyes developed a macular hole with retinal detachment at 16 months after ILM peeling [5]. Similarly in another study, among 15 eyes, macular hole was seen in two eyes after ILM peeling [6]. Our cases both describe exudative diseases (hemorrhage and optic pit maculopathy), which occur with a different mechanism than myopic foveoschisis. The caution about peeling however is pertinent to all those situations. In cases where ILM peeling is still deemed necessary, fovea-sparing ILM peeling can reduce the risk of macular hole formation [7, 8]. Intraoperative SDOCT may identify occult FTMH and help determine the need for tamponade and postoperative positioning for hole closure [9].

Vitreoretinal surgeons may wish to consider “omega sign” in the process of their surgical decision making and surgical planning. This may reduce the threshold for ILM peeling in these patients.
